# mRNA Lipoplexes with Cationic and Ionizable α-Amino-lipophosphonates: Membrane Fusion, Transfection, mRNA Translation and Conformation

**DOI:** 10.3390/pharmaceutics14030581

**Published:** 2022-03-07

**Authors:** Sohail Akhter, Mathieu Berchel, Paul-Alain Jaffrès, Patrick Midoux, Chantal Pichon

**Affiliations:** 1 Centre de Biophysique Moléculaire, CNRS UPR4301, Rue Charles Sadron, CEDEX 02, 45071 Orléans, France; sohail.akhter@tevaruncorn.co.uk (S.A.); patrick.midoux@cnrs.fr (P.M.); 2 Région Centre-Val de Loire, Le Studium Loire Valley Institute for Advanced Studies, 9 Rue Saint-Pierre Lentin, 45000 Orléans, France; 3 CNRS, CEMCA, UMR 6521 CNRS, Université de Bretagne Occidentale, 6 Avenue Victor Le Gorgeu, 29238 Brest, France; mathieu.berchel@univ-brest.fr (M.B.); paul-alain.jaffres@univ-brest.fr (P.-A.J.)

**Keywords:** mRNA transfection, cationic liposomes, lipoplexes, α-aminolipophosphonate, imidazolium lipophosphoramidate, dendritic cells

## Abstract

Cationic liposomes are attractive carriers for mRNA delivery. Here, mRNA lipoplexes (LX) were prepared with the cationic lipids α-aminolipophosphonate (**3b**) or imidazolium lipophosphoramidate (**2**) associated with various α-aminolipophosphonates co-lipids comprising protonable groups (imidazole or pyridine) and **DOPE**. Physicochemical parameters of liposomes and their membrane fusion activity were measured. LXs comprising either **3b**- or **2**- allowed transfection of ~25% and 40% of dendritic cells with low cytotoxicity, respectively; the efficiency increased up to 80% when **2** was combined with the imidazole-based co-lipid **1**. The transfections were high with **3b**/**1**, **3b**/**DOPE**, **2**/**1** and **2**/**DOPE** LXs. We observed that the transfection level was not well correlated with the acid-mediated membrane fusion activity of liposomes supposed to destabilize endosomes. The mRNA release from LXs and its translation capacity after release were studied for the most efficient LXs. The results showed that the more mRNA was condensed, the poorer the translation efficiency after release was. In contrast to DNA, circular dichroism performed on mRNA complexed with **2**/**DOPE** revealed the presence of denatured mRNA in LXs explaining this lack of translation efficiency. This is an important parameter that should be stressed for the preparation of mRNA LXs with a conserved mRNA translation activity.

## 1. Introduction

**Figure 1:**
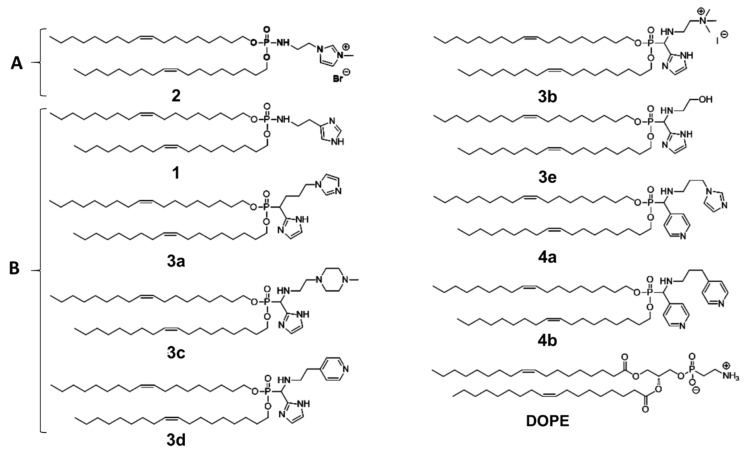
Molecular structure of lipids used in this work (**A**) cationic lipids and (**B**) co-lipids.

Today, the effectiveness of mRNA vaccines is demonstrated through the development and use of approved vaccines for the COVID-19 virus [1,2,3,4,5,6]. This efficiency now makes it possible to consider using synthetic mRNAs for multiple therapeutic applications such as viral infections, cancer, gene therapy, cell reprogramming, and genome editing (CRISPR/Cas9) [7,8,9,10,11,12,13,14,15,16,17,18]. For these purposes, mRNA delivery systems are required to protect mRNA against nucleases and ultimately to deliver mRNA to the target cells in vivo, to release it intracellularly and to allow for its efficient translation. For vaccines, lipid nanoparticles (LNP) have demonstrated efficacy to protect synthetic mRNA or self-amplifying mRNA from RNAse degradation and increase their cellular uptake upon intramuscular injection. LNP formulations consisting of (i) one ionizable lipid or polymer containing tertiary amines featuring a pKa < 6.5; (ii) one amphoteric lipid such as 1,2-distearoyl-*sn*-glycero-3-phosphocholine (DSPC) mimicking the lipid membrane in cells; (iii) cholesterol; and (iv) one polyethylene glycol (PEG)-lipid conjugate. Besides LNP, cationic liposomes remain attractive vectors for mRNA delivery. For all formulations, the cytosolic delivery of mRNA is a crucial step. Lipoplexes (LX) are mostly prepared by the association of a cationic lipid and a co-lipid. Cationic lipids allow electrostatic interactions with nucleic acids while the co-lipids contribute to reduce cytotoxicity and induce the destabilization of the endosomal membrane that favors mRNA transfer in the cytosol [19]. **DOPE** (1,2-Dioleoyl-*sn*-glycero-3-phosphoethanolamine) is the most common co-lipid used because it has the propensity to induce favorable supramolecular changes (from lamellar to inverted hexagonal supramolecular assemblies), thus promoting membrane fusion in a mild acid environment typical of the endosome lumen [20,21,22,23,24]. Likewise, lipids containing histidine or imidazole polar head group promote the endosomal escape of the nucleic acid through protonation of imidazole moiety in the acid lumen of endosomal vesicles by contributing to the proton-sponge phenomena and/or the membrane fusion effect [25,26,27]. Liposomes made with an equimolar ratio of the *O*,*O*-dioleyl-*N*-(3-*N*-(*N*-methylimidazolium iodide)propylene) phosphoramidate (**2**) and *O*,*O*-dioleyl-*N*-histamine phosphoramidate (**1**) were successfully used for in vitro DNA and RNA transfection with negligible cytoxicity [28,29] and for in vivo transfection in association with cationic polymers (Figure 1) [30,31,32].

Here, we evaluated mRNA transfection with cationic liposomes comprising either the lipid **2** or the α-amino-lipophosphonate featuring tertiary amine (**3b**) as cationic lipids associated with either **DOPE** or pH responsive α-amino-lipophosphonate lipids as co-lipids (Figure 1) [33]. The membrane fusion activity of liposomes were measured at pH 7.4 and pH 5.5 and transfection efficiencies of lipoplexes with mRNA encoding EGFP were evaluated in the murine dendritic DC2.4 cell line. The mRNA release capacity and its post-release translational efficiency were investigated for the most efficient LXs. Conformation of mRNA within 2/DOPE lipoplexes was determined. Lastly, mRNA transfection was compared with plasmid DNA transfection.

## 2. Materials and Methods

All the chemicals used in this study were purchased from Sigma (St. Quentin Fallavier, France) unless otherwise stated. Phosphate-buffered saline (PBS) was supplied by PAA Laboratories (Les Mureaux, France). *O*,*O*-dioleyl-*N*-histamine phosphoramidate (**1**) and *O*,*O*-Dioleyl-*N*-(3-*N*-(*N*-methylimidazolium iodide)propylene) phosphoramidate (**2**) were synthesized as previously described [25,26]. All α-amino-lipophosphonates were synthesized as previously described [33].

### 2.1. In Vitro Transcribed mRNA

Synthetic mRNA-EGFP and mRNA-Luc were produced as previously described [34]. pGEM4Z-EGFP-A64 and pGEM-4Z-Luc-A64 plasmids were linearized with SpeI. Linearized plasmids were used as a template for in vitro transcription using the mMessage mMachine T7 Transcription Kit (Ambion, Oxfordshire, UK) to produce anti-reverse cap analogue (ARCA)-A100 mRNAs. Synthetic mRNA-EGFP and mRNA-Luc had an ARCA modified cap and a 100-adenosine poly(A) tail. The RNA concentration was determined by measuring the absorbance at 260 nm; RNA had 260:280 ratios ≥2 and was stored at −80 °C in small aliquots. IVT mRNA-Luc was labelled with the fluorescein Label IT kit from MIRUS (Madison, WI, USA) and purified by ethanol precipitation.

### 2.2. Plasmid DNA (pDNA)

pCMV-EGFP (5130 bp) was homemade plasmid DNA encoding the jellyfish Aequorea victoria enhanced green fluorescent protein (EGFP) under the control of the human cytomegalovirus (CMV) promoter. Supercoiled pDNA was isolated from Escherichia coli DH5 at supercompetent bacteria (Invitrogen, Cergy Pontoise, France) by alkaline lysis and purification was performed by using a QIAGEN EndoFree Plasmid Mega Kit (Qiagen, Courtaboeuf, France).

### 2.3. Cells and Cell Culture

The murine dendritic cell line (DC2.4 cells) was kindly given by Dr P. Jeannin and Dr Yves Delneste (INSERM UMR 892 Nantes-Angers, France) [35]. They were grown at 37 °C in a humidified atmosphere that contains 5% CO_2_ in Dulbecco’s modified Eagle’s medium (PAA Laboratories) supplemented with 10% fetal calf serum (PAA Laboratories) with 100 units per mL of antibiotics penicillin and 100 mg per mL of streptomycin (PAA Laboratories). The mycoplasma-free condition of the cells was evidenced using the MycoAlerts Mycoplasma Detection Kit (Lonza, Levallois Perret, France).

### 2.4. Liposomes

Liposomes were prepared by the film hydration method followed by sonication. For this, we used the required volume of a 3.5 mM or 1.75 mM co-lipid to 3.5 mM cationic lipid in either equimolar or 2/1 cationic/co-lipid ratio with absolute ethanol to the total volume of 500 mL in a round bottle flask. The solution was evaporated under vacuum in the rotary evaporator until a dry film remained at the base of the flask. The film was further hummed with nitrogen followed by the hydration with 10 mM HEPES buffer (pH 7.4) overnight at 4 °C. Finally, the dispersion was sonicated for 10 min at the room temperature at 37 kHz using a bath sonicator (Fischer Bioblock Scientific, Illkirch, France).

### 2.5. Lipoplexes (LX) Preparation

LX were prepared by upside down pipetting of mRNA or pDNA to liposomes at mRNA or DNA/liposomes charge ratio of 1/0.5, 1/1 and 1/2. For this, 2.5 µg mRNA or pDNA at 1 mg/mL were added to 0.75 µL, 1.5 µL and 3 µL of 5 mM liposomes in 10 mM HEPES buffer, pH 7.4, and the solution was kept stationary for 30 min at room temperature before being diluted in 0.5 mL of serum-free culture medium for transfection. Agarose gel (0.6% *w*/*v*) electrophoresis was performed to affirm the complex formation of mRNA or pDNA with liposomes at these ratios.

### 2.6. Size Distribution and ζ Potential Measurements

Liposomes and LX size distribution and ζ potential were measured at 25 °C on an SZ-100 Analyser (Horiba Scientific, les Ulis, France). For the analysis, the samples were prepared in cuvettes by adding 50 µL of the liposomes or LX solution to 1.45 mL of 10 mM HEPES buffer, pH 7.4. Size distribution by the hydrodynamic diameter was determined by dynamic light scattering using the photon correlation spectroscopy technique and calculated from the size distribution by volume (generated by the Stokes–Einstein equation for polydisperse samples), provided by the inbuilt software, and are reported as the average of three independent measurements ± the deviation from the mean. The uniformity of size distribution was recorded as the polydispersity index (PDI) obtained with the particle size. The ζ potential, the electrophoretic mobility of liposome dispersion, was recorded on the same instrument by using the zeta mode to monitor the global surface charge.

### 2.7. Membrane Fusion Study

The membrane fusion potential of liposomes was measured using the fluorescence resonance energy transfer (FRET) assay as described [36]. *N*-(7-nitro-2-oxa-1,3-diazol-4-yl)-1,2-dihexadecanoyl-sn-glycero-3-phosphoethanolamine (NBD-PE;) (Thermo Fisher Scientific, Les Ulis, France) and Rhodamine linked to 1,2-dihexadecanoyl-*sn*-glycero-3-phosphoethanolamine (Rho-PE; Thermo Fisher Scientific) were used as donor and acceptor fluorescent lipids, respectively. Fluorescent liposomes showing FRET phenomena were prepared as follows; PC (egg yolk phosphatidylcholine) (1 mmol), NBD-PE (0.05 mmol) and Rho-PE (0.05 mmol) dissolved in chloroform were dried under reduced pressure. The dried lipid film was hydrated overnight at 4 °C in 2 mL of 10 mM HEPES buffer, pH 7.4. The suspension was vigorously vortexed for 2–5 min at room temperature and further sonicated for 15 min in a cold bath sonicator at 37 kHz (Bioblock Scientific, Strasbourg, France). Fusion was induced directly in the cuvette by adding 20 µL aliquots of NBD-PE/Rho-PE liposomes (0.01 mmol) to tested liposomes (100 µL at 5.4 mM) either in 10 mM HEPES buffer, pH 7.4 or 10 mM HEPES solution, pH 5.5. All experiments were performed at room temperature. The fluorescence intensity was measured 15 min after aliquot addition upon excitation at 465 nm and emissions at 530 and 580 nm for NDB and Rho, respectively. All measurements were done at room temperature. The maximum of fusion (I_max_) was determined from the rhodamine fluorescence intensity of PC/NBDPE/Rho-PE liposomes in the presence of 0.1% TritonX100 and the fusion ability of the test samples was analyzed by using the formula: % Fusion (t) = [(I_(t)_ − I_0_)] × 100/[(I_max_ − I_0_)] where % Fusion (t) indicates the percentage of fusion at different time points and I and (t) are fluorescence intensity at 580 nm and the time period respectively. I_0_ corresponds to the fluorescence intensity of liposomes at 580 nm in the absence of test samples.

### 2.8. Transfection

Transfection was evaluated on the murine DC line (DC2.4 cells), one established clone from bone marrow of C57BW6 mice cultured in GM-CSF [37]. The cells were grown at 37 °C in a humidified atmosphere containing 95% air and 5% CO_2_ in DMEM supplemented with 10% fetal calf serum (PAA Laboratories, Les Mureaux, France), 100 Units/mL of penicillin and 100 mg/mL of streptomycin (Fischer Bioblock, Illkirch, France). Two days before transfection, cells were seeded in 24 well culture plates at a density of 1 × 10^5^ cells per cm^2^. At the time of transfection, cells were 80% confluent. The culture medium was discarded; cells were washed with serum-free medium and then incubated for 4 h at 37 °C with LX (2.5 µg of mRNA or pDNA diluted in 500 mL serum-free culture medium). Afterwards, the medium was removed and replaced with fresh medium containing 10% serum and cultured at 37 °C without any LX for 24 h and 48 h in the case of mRNA and DNA transfection, respectively. Finally, the cells were washed twice with PBS, harvested with trypsin, centrifuged (800× *g* for 5 min at 4 °C) and suspended in PBS. EGFP expression was quantified by flow cytometry (LSR, Becton Dickinson, Grenoble, France) by measuring the cell-associated fluorescence intensity at ƛ_ex_ = 488 nm and ƛ_em_ = 520 ± 24 nm.

### 2.9. Cytotoxicity Assay

48 h after transfection, 3-(4,5-dimethylthiazol-2-yl)-2,5-diphenyltetrazolium bromide (MTT; 100 mL of 5 mg per mL solution in PBS) was added to each well followed by incubation at 37 °C for 4 h. MTT converted in Formazan was solubilized with acidic isopropanol and quantified by measuring the absorbance at 570 nm with a spectrophotometer. Cell viability was calculated for each treatment as ‘‘absorbance of LX treated cells/absorbance of control cells’’ × 100. Absorbance measured for the non-transfected cells cultured under the same conditions as those of transfected cells was used for the control cells. The results were presented as the mean of three experiments with each measurement in triplicate.

### 2.10. Laser Scanning Confocal Microscopy

Fluorescein labelled mRNA-LXs were observed by using a Zeiss Axiovert 200 M microscope coupled with a Zeiss LSM 510 Meta scanning device (Carl Zeiss Co., Ltd., Iena, Germany). The inverted microscope was equipped with a Plan-Apochromat 63× objective (NA ¼ 1.4) and with a temperature-controlled stage. Cells were seeded at 1 × 10^4^ cells per wells in 0.5 mL of culture medium in a 4-well Lab-Tek chambered coverglass (Nunc, Dutsher S.A., Brumath, France). The day of transfection, cells were washed three times in a serum-free medium. Then, 0.5 mL of LX with 2.5 µg fluorescein labelled mRNA in serum-free medium was added and cells were incubated for 2 h at 37 °C. Then, cells were fixed in 3% *p*-formaldehyde in cold PBS.

### 2.11. Reticulocyte Assay

In vitro (cell-free) luciferase activity was tested using a Rabbit Reticulocyte Lysate System, Nuclease treated (Promega, Madison, WI, USA) following the manufacturer’s instruction. In 1.5 mL tube, 2.6 µL HEPES buffer (10 mM, pH 7.4) containing 0.5 µg of synthetic mRNA-Luc free, complexed with LX or upon DS treatment of LX were adjusted to the total volume of 7µL, and then mixed with 18µL of reticulocyte mixture and incubated for 1 h 30 min at 30 °C. Finally, 2.5 µL of this mixture was added to 50 µL of LAR (Luciferase Assay Reagent) and the luciferase activity was measured two times for 10 s with a 2 s interval using a luminometer (LUMAT LB 9507, Berthold, Wildbach, Germany).

### 2.12. Circular Dichroism Spectroscopy (CD)

CD spectra were recorded on a Jasco J-715 spectropolarimeter equipped with a Peltier device for the temperature control operating at 1.0 nm resolution. Quartz cells of suitable path length were used. Spectra are the average of at least four runs, performed in the range of 220–330 nm. All the spectra were normalized by the nucleotide concentration previously determined by absorption spectroscopy. CD spectra were normalized and reported as molar circular dichroism, Δε (M^−1^ cm^−1^).

## 3. Results and Discussion

### 3.1. Liposomes Physicochemical Properties

The physicochemical characteristics of lipid **3b**-based liposomes are presented in Table 1. The average size was 147 nm (0.1< PDI < 0.5), the formulation **3b**/**3e** featured the smallest size (85 nm) while the **3b**/**3a** liposomes had the biggest size (235 nm). Their ζ potential averaged at 69 mV; **3b**/**3a** (49 mV) and **3b**/**3c** (56 mV) being less positively charged. For lipid **2**-based liposomes, they had a size distribution of 104 nm in average (0.1< PDI < 0.5) with the biggest one at 207 nm (**2**/**DOPE**) and two intermediates at 148 nm (**2**/**3d**) and 163 nm (**2**/**4b**) (Table 2). Their ζ potential averaged at 64 mV, the more positively charged being **2**/**1** at 81 mV.

For the series **3b**, the most fusogenic liposomes at acidic pH were those including co-lipids **3a** or **1** (Table 3). The presence of an imidazole group close to the phosphonate of the co-lipid is not a determining factor when associated with lipid **3b**. Other **3b**-liposomes with co-lipids **3c** or **4a** featured an intermediate fusogenic behaviour. Finally, **3b**-liposomes associated with co-lipids **3d**, **3e**, **4b** or **DOPE** were not or only weakly fusogenic. Of note, the presence of a terminal pyridine as polar head group (**3d** and **4b**) did not promote membrane fusion both at neutral and acidic pH. Overall, the presence of an imidazole in the head group of co-lipids of α-amino-lipophosphonates featuring two ionizable aza-heterocycles (imidazole, **3a** or pyridine, **4a**) induced membrane fusion at pH 5.5. Liposomes of the series **2** were not or weakly fusogenic at neutral pH. Only liposomes with co-lipids **1** or **DOPE** were highly fusogenic at pH 5.5 which is the pH of the lumen of late endosomes. To sum-up, the presence of an imidazole group close to the phosphonate did not promote membrane fusion when associated with lipid **2**. Moreover, the association of DOPE with lipid **2** and **3b** at a lipid molar ratio of 1/1 and 2/1 increased the fusogenicity of liposomes at pH 5.5.

### 3.2. mRNA Transfection

**Figure 2:**
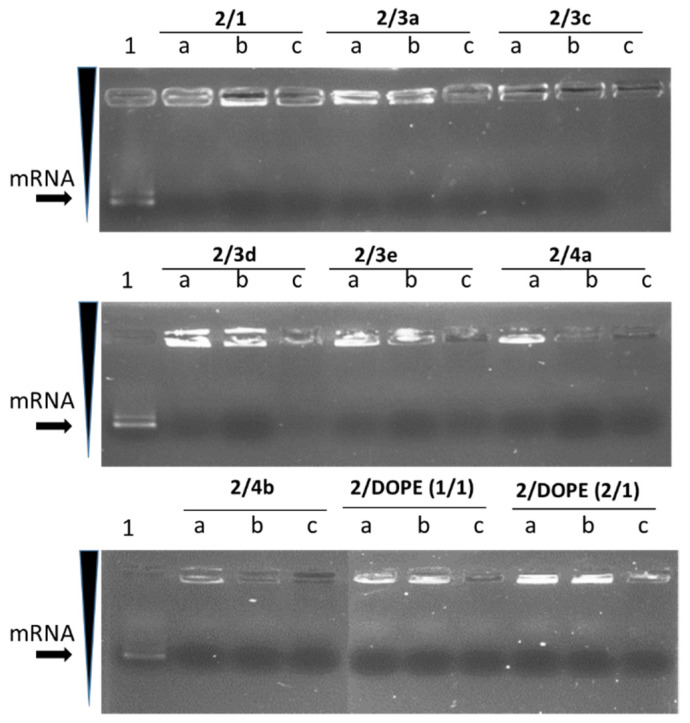
Agarose gel electrophoresis of LXs. mRNA was complexed with **2**-based liposomes at mRNA/liposomes charge ratios of 1/0.5 (a), 1/1 (b) and 1/2, (c). Lane 1 was mRNA in the absence of liposomes. Then, LX were analyzed on a 0.6% agarose gel. Gel running buffer was TAE (40 mM Tris-acetate, 1 mM EDTA). mRNA gel was stained with ethidium bromide (EtBr) at a concentration of 0.5 μg/mL. mRNA fluorescence was revealed under UV lamp.

It is well known that the charge ratio of mRNA to liposomes (mRNA/L) directly influences the transfection efficiency of LX. Therefore, optimization of this ratio is crucial to find out the right balance between the efficacy and the cytotoxicity. Thus, in vitro transcribed (IVT) mRNA encoding EGFP was complexed with liposomes at three different mRNA/L ratios and used to transfect murine dendritic DC2.4 cells. Figure 2 shows an agarose gel electrophoresis of mRNA complexed with **2**-based liposomes at different ratios.

For all tested mRNA/L ratios, the mRNA was quantitatively complexed with liposomes and no migration of free mRNA was observed (Figure 2). The brightness of mRNA in the well decreased when the strength of the mRNA condensation in LX increased in line with liposomes quantity. Similar observations were found with mRNA complexed with **3b**-based liposomes (data not shown).

**Figure 3:**
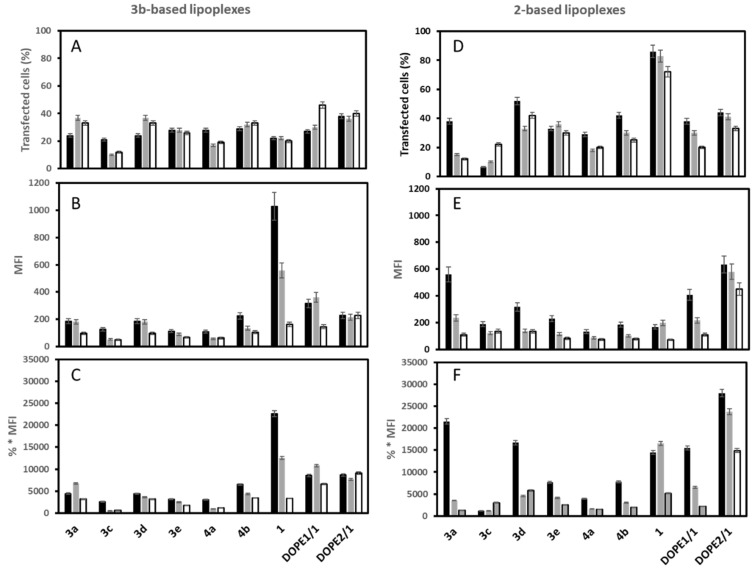
Transfection efficiency of mRNA lipoplexes. DC 2.4 cells were transfected for 4 h with lipoplexes made eGFP mRNA at (black bars) 1/0.5, (grey bars 1/1) and (white bars) 1/2 mRNA/L charge ratio. The expression of eGFP was measured 48 h post-transfection by flow cytometry. (**A**,**D**) Transfected cell (%) corresponds to the percentage of eGFP positive cells; (**B**,**E**) MFI is the mean of the fluorescence intensity related to the amount of eGFP produced by the cell; (**C**,**F**) % * MFI is the global transfection efficiency (i.e., the product of the number of EGFP positive cells by MFI). Values are means ± SD of three different experiments.

The transfection outcomes with the **3b**-LXs indicated that the percentage of transfected cells with **3b**/**3e**, **3b**/**4a**, **3b**/**4b**, **3b**/**DOPE** LXs averaged around 30%, and the high of 40% was obtained with the two **3b**/**DOPE** LXs, while it reached only 22% for **3b**/**3a**, **3b**/**3c**, **3b**/**3d** and **3b**/**1** LXs (Figure 3C). The mRNA translation was quite low (MFI < 200) except for **3b**/**1** LX, which led to a high MFI value with the level being inversely proportional to the mRNA/L ratio (1000, 800 and 200 at 1/0.5, 1/1 and 1/2 mRNA/L ratio, respectively). Overall, formulations made with **3b** and co-lipids **3d** (one imidazole close to the phosphonate and a polar head with a terminal pyridine), **4b** (one pyridine close to the phosphonate and a polar head with a terminal pyridine) and **DOPE** were not very efficient in terms of translation when compared to the **3b**-LXs prepared with the co-lipid **1** (polar head with a terminal imidazole).

The transfection outcome with **2**-based LXs indicated that the highest transfection efficiencies were obtained at mRNA/L ratio of 1/0.5. Only a few formulations had a positive impact on the number of transfected cells (average around 40%; the highest with **2**/**1** LX being at 86%) and on the EGFP mRNA translation (MFI) level (Figure 3D,E). Overall, **2**-based LXs allowed a higher number of transfected cells than the **3b**-based LXs. For **2**-based LX, high transfections were obtained with co-lipids **3a** (an imidazole close to the phosphonate and a polar head with a terminal imidazole), **3d** (one imidazole close to the phosphonate and a polar head with a terminal pyridine), **1** (a polar head with a terminal imidazole) and **DOPE**.

The cationic lipid **3b** having one imidazole close to the phosphonate and one terminal quaternary ammonium seems to accept only co-lipids that do not have a heterocycle (imidazole, pyridine) close to the phosphonate moiety. Conversely, the cationic lipid **2** accommodates co-lipids with imidazole close to the phosphonate and imidazole (**3a**) or pyridine (**3d**) at the terminal group.

The percentages of cell viability upon transfection with **3b**- and **2**-based LXs are shown in Table 4. Transfection with **3b**-based LXs was weakly cytotoxic as indicated by the high percentage of viable cells (>80%) except for **3b**/**3e** LX that resulted in ~30% of dead cells at an mRNA/L ratio of 1/1. It is worth noting that transfection with **3b**/**1** LX, the most efficient LX, resulted in 83 ± 6 and 100 ± 8% of viable cells at mRNA/L ratio of 1/0.5 and 1/1, respectively. For the **2**-based LXs, cell viabilities depended on the nature of the co-lipid and on mRNA/L ratios. At mRNA/L ratio of 1/0.5, most of **2**-based LX were not cytotoxic too (>80% viable cells), except for **2**/**4b** and **2**/**1** LXs. **2**/**1** and **2**/**DOPE** LXs showed high transfection efficacies. At an mRNA/L ratio of 1/1, **2**/**1** LX was more cytotoxic-~30% of viable cells-than at a ratio 1/0.5. In contrast, cytotoxicity remained low with **2**/**DOPE** LX at ratio 1/1. The cytotoxicity of **2**/**1** LX could come from LX *per se* or free **2**/**1** liposomes.

**Figure 4:**
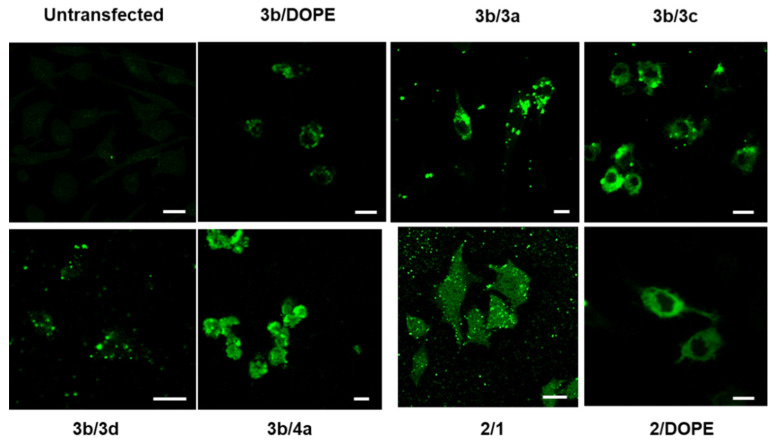
Confocal microscopy analysis of upon transfection mRNA-LX. DC2.4 cells were transfected for 2 h with 2.5 μg of fluorescein labelled IVT mRNA complexed with the indicated liposomes at mRNA/L 1/0.5. In **3b**/**DOPE** and **2**/**DOPE** LX, the lipid molar ratio was 2/1. Scale bars = 25 µm.

For **3b**-based liposomes, the most efficient in terms of transfection were obtained with liposomes **3b**/**1** and the two **3b**/**DOPE**. **3b**/**1** and **3b**/**DOPE** (2/1) have a high membrane fusion potential at pH 5.5 of 71% and 58%, respectively. Despite the high membrane fusion potential of **3b**/**3a** liposomes (79%, at pH 5.5), the corresponding LX did not promote high transfection. Among **2**-based liposomes, the most efficient in terms of transfection were liposomes **2**/**3a**, **2**/**3d**, **2**/**1** and **2**/**DOPE**, while the most fusogenic were **2**/**1** and **2**/**DOPE** liposomes (Table 3). These results suggest that liposomes inducing more than 60% of membrane fusion at pH 5.5 lead to an interesting global transfection efficiency as observed for the formulations **3b**/**1**, **2**/**1**, and **2**/**DOPE**. LXs made with **2**/**3a** and **2**/**3d** liposomes that are not or weakly fusogenic at pH 5.5 promote high transfection when used at mRNA/L ratio of 1/0.5. Altogether, these data indicate the absence of direct correlation between the transfection efficiencies and the liposome fusion properties. This is in line with the fact that the fusogenic potential of liposomes is likely impacted by its interaction with the mRNA. Fluorescence microscopy experiments were performed to observe the intracellular fate of mRNA upon delivery. Figure 4 shows confocal microscopy images recorded 2 h after incubation of the cells with LXs made with fluorescein-labelled IVT mRNA at mRNA/L 1/0.5 (the ratio giving the highest transfection efficiency as shown in Figure 3). Representative images of LXs made with fusogenic and non-fusogenic **3b** liposomes were presented. For 2-series, only LXs with the most acid-fusogenic **2**/**1** and **2**/**DOPE** liposomes are shown in this figure. Efficient mRNA delivery in the cytosol was evidenced by the presence of high diffuse fluorescence in the cytosol. This was observed for **3b** LXs containing **3a**, **3c**, **3e**, **4a** or **DOPE** as co-lipids as well as for **2**/**1** LX. By contrast, the fluorescence staining with **3b/d** was more compacted (vesicular) and less diffuse in the cytosol. The high acid-mediated membrane fusion of **3b**-liposomes with co-lipids **3a** (79%), **3c** (41%), **4a** (37%) and **DOPE** (58%) as well as liposomes **2**/**1** (97%) suggested that it contributed to endosome escape after endocytosis.

In addition to endosomal escape, the mRNA must be released in an intact structure after the compaction by liposomes to be efficiently translated by ribosomes. Therefore, we investigated the mRNA release, translation capacity of post-release mRNA. Moreover, the mRNA conformation in the presence of liposomes was assessed. These investigations were conducted with **2**/**1** and **3b**/**1** liposomes (lipid molar ratio of 1/1) and **2**/**DOPE** and **3b**/**DOPE** liposomes (lipid molar ratio 2/1) which showed good transfection. **3b**/**DOPE** and **2**/**DOPE** LXs exhibited a similar size of 220 ± 10 nm and 200 ± 10 nm, respectively. **3b**/**DOPE** LX was less positive than **2**/**DOPE** with a ζ potential of 17 ± 1 mV and 33 ± 2 mV, respectively. **3b**/**1** LX were larger and less positive (260 ± 15 nm; 20 ± 1 mV) than **2**/**1** LX (160 ± 9 nm; 27 ± 2 mV).

### 3.3. mRNA Release from LX

**Figure 5:**
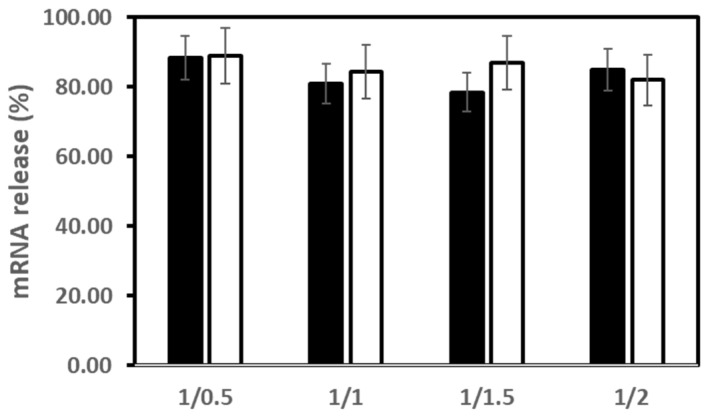
mRNA release from LX. mRNA was first complexed with (black bar) **2**/**DOPE** (lipid molar ratio 2/1) or (white bar) **3b**/**DOPE** (lipid molar ratio 2/1) liposomes at various mRNA/L ratio. Then, LX were incubated in the absence or the presence of 90 µM dextran sulphate of 500 kDa (DS) for 1 h before electrophoresis on a 0.6% agarose gel. Gel running buffer was TAE (40 mM Tris-acetate, 1 mM EDTA) and mRNA gel was stained with ethidium bromide (0.5 μg/mL). mRNA fluorescence was revealed under UV lamp and quantified by image J. The percentage of mRNA release was determined from the intensity of mRNA that migrated after DS treatment of LX and the intensity of the same amount of free mRNA.

First, the release of mRNA from LXs incubated in the presence of dextran sulphate (DS) as polyanions was assessed by agarose gel shift assay. For **2**/**DOPE** and **3b**/**DOPE** LXs, there was a high release of mRNA (80–90% of mRNA) from LX upon incubation in the presence of 90 µM DS whatever the mRNA/L ratio used (Figure 5).

### 3.4. Translation Capacity Post-Release mRNA

**Figure 6:**
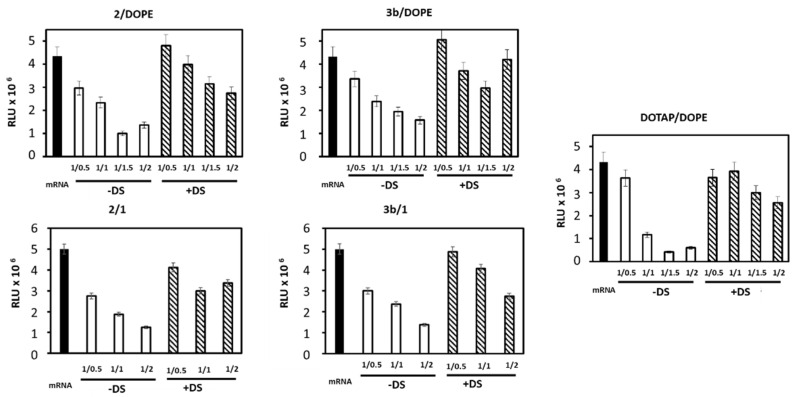
In vitro transcription of mRNA-Luc LX. mRNA-Luc (0.2 μg) (black bar) free or complexed with the indicated liposomes at various mRNA/L ratio was incubated (white bar) in the absence or (hacked bar) the presence of 90 µM DS for 1 h. For **2**/**1** and **3b**/**1** LXs the lipid molar ratio was 1/1; for **2**/**DOPE** and **3b**/**DOPE** LX it was 2/1 and it was 1/1 for **DOTAP**/**DOPE**. Next, samples were mixed with the TNT Quick Coupled Transcription/Translation Systems Kit (Promega). After 1 h 30 min incubation at 30 °C, the luciferase activity was measured and expressed as relative light unit (RLU).

Next, in vitro transcription of mRNA was performed with mRNA coding luciferase complexed with **2**/**1**, **2**/**DOPE**, **3b**/**1**, **3b**/**DOPE** and also **DOTAP**/**DOPE** liposomes as reference. The experiments were conducted as a function of mRNA/L ratio before and after LX destabilization (i.e., mRNA release) in the presence of DS, as done above. Compared to free mRNA, the luciferase activity was impaired with the increased liposomes quantity (Figure 6). The decrease was 60% and 57% for **2**/**1** and **2**/**DOPE**, respectively; it was 53% and 42% for **3b**/**1** and **3b**/**DOPE** LXs at mRNA/L ratio 1/1. It looked less drastic than with **DOTAP**/**DOPE** LX (76%) at the same ratio. When LXs were treated with DS before in vitro transcription, the luciferase activity was partially recovered. The recovery was quasi complete at ratio 1/0.5 with **2**/**1**, **2**/**DOPE**, **3b**/**1** and **3b**/**DOPE** LXs. Despite the efficient release of mRNA from both LXs, their transfection efficiency was different. **2**/**DOPE LXs** were more efficient than **2**/**1**, **3b**/**1** and **3b**/**DOPE** ones; **2**/**1** and **3b**/**1** were close (Figure 3). This difference could be due to the impact on the mRNA structure after interaction with liposomes. Indeed, the condensation of the mRNA could induce an irreversible denaturation of a part of mRNA, thus affecting its translation efficiency. We hypothesize that such a denaturation would increase when the amount of liposomes used in the formulation increased.

### 3.5. mRNA Conformation

**Figure 7:**
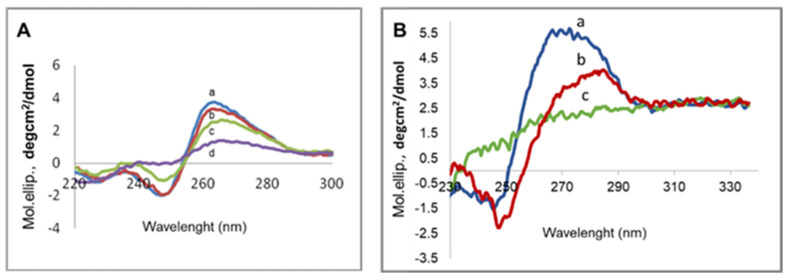
Circular dichroism of LX. (**A**) (a, blue) mRNA. mRNA complexed with **2**/**DOPE** (lipid molar ratio 2/1) liposomes at mRNA/L ratio 1/0.5 (b, red), 1/1 (c, green) and 1/2 (d, purple). (**B**) (a, blue) pDNA. pDNA complexed with **2**/**DOPE** (lipid molar ratio 2/1) liposomes at DNA/L ratio 1/1.5 (b, red)1/2, (c, green).

To assess the occurrence of mRNA denaturation, we performed CD measurements of mRNA in the absence and the presence of increasing quantity of **2**/**DOPE** liposomes (Figure 7A). The CD spectrum of free mRNA showed a positive band at 222 nm with a shoulder at 235 nm, a negative minimum near 248 nm and positive peak at 267 nm. This CD spectrum was typical to mRNA in the A-form double helix conformation to single stranded polynucleotides containing base stacking regions forming a double helix [37,38,39]. After complexation with the cationic liposomes, there is a decrease of the molar ellipticity of the negative minimum near 248 nm and the positive peak at 267 nm, concomitant with a small red shift, which increased with the mRNA/liposome ratio indicative of mRNA condensation.

This conformational change in the presence of cationic liposomes was similar to that reported for mRNA with POPC/DOTAP liposomes showing a drastic denaturation of mRNA and a loss of translational activity [38]. Thus, we observed a nice correlation between CD spectra and protein expression in reticulocyte and transfection, which indicates that the compaction of mRNA during LX formation induced in turn an irreversible conformational change in mRNA, the so-called A-form [38,40], leading to decreased translation efficiency after intracellular release. To confirm this effect, a study should be carried out for other types of liposomes. 

Last, we investigated the conformation of pDNA by CD spectra measurement in the absence and in the presence of increased quantity of **2**/**DOPE** liposomes (Figure 7B). The CD spectrum of pDNA was characteristic to CD of B-type DNA with a negative band at 242 nm and a positive peak around 273 nm. Following the addition of cationic liposomes, the positive peak showed a marked intensity decrease accompanied by a strong red shift toward 283 nm while disappearing at a high DNA/liposomes ratio. The negative band at 242 nm progressively decreased and completely disappeared at the high DNA/liposomes ratio. These changes resulted from DNA compaction without denaturation when the amount of liposomes increased and were comparable to CD of calf-thymus DNA in the presence of DOTAP/DOPE liposomes. These changes resulted from DNA compaction without denaturation when the amount of liposomes increased and were comparable to CD of calf-thymus DNA in the presence of DOTAP/DOPE liposomes [41]. This is an important point to consider for all LXs.

### 3.6. mRNA Transfection versus DNA Transfection

**Figure 8:**
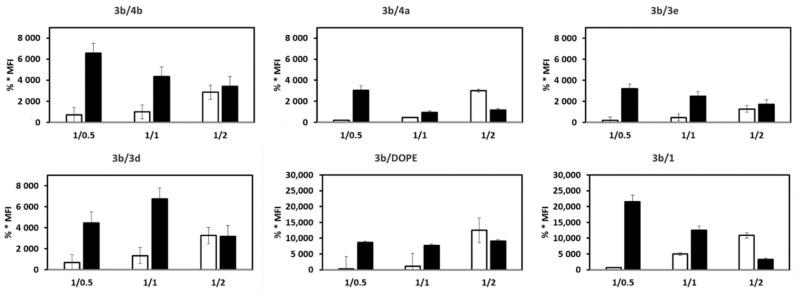

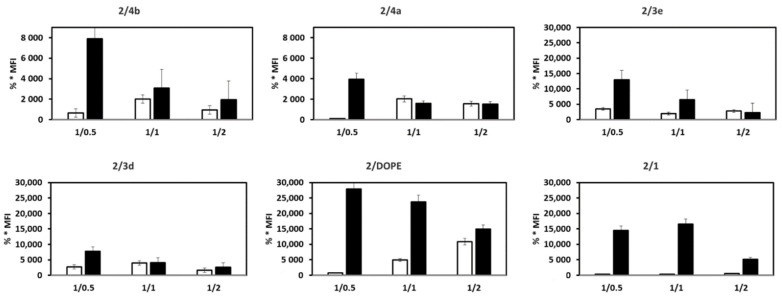
Comparison between the transfection efficiency of mRNA and DNA LXs. DC2.4 cells were transfected with 5 μg of (black bar) mRNA-EGFP or (white bar) pEGFP complexed with the indicated liposomes at indicated mRNA/L or DNA/L ratio. In **3b**/**DOPE** and **2**/**DOPE** LX, the lipid molar ratio was 2/1. % * MFI is the global transfection (i.e., the product of the number of EGFP positive cells by MFI). The final values are means ± SD of three different experiments.

Next, we compared the transfection efficiency of mRNA vs. pDNA as a function of liposomes and mRNA/L or pDNA/L ratio (Figure 8). **3b**/**4b**, **2**/**4b**, **3b**/**4a**, **2**/**4a**, **3b**/**3e**, **2**/**3d**, **2**/**DOPE** and **3b**/**1** LXs allowed higher mRNA transfection at ratio 1/0.5. **3b**/**3d** LX allowed better mRNA transfection at ratio 1/1. Overall, pDNA transfection was efficient at ratio 1/2 or 1/1, while that of mRNA transfection was at a ratio 1/0.5. In addition, the transfection level was often higher with mRNA than with DNA. For comparison, when lipofectamine (LFM) was used as the standard transfecting reagent, the global transfection (% * MFI) values were 28,138 and 8463 for mRNA and DNA transfection, respectively. This was probably related to their respective location of expression machinery. The pDNA must remain condensed until it reaches the nuclear envelope and released into the nucleus. Conversely, mRNA must be released into the cytosol and available to the ribosome machinery for translation. Thus, lipoplexes should be less stable with mRNA than with pDNA.

## 4. Conclusions

This study reveals that the cationic α-aminolipophosphonate (**3b**) containing a tertiary aliphatic amine in the polar head group allows the efficient transfection of mRNA when formulated with either co-lipid **1** or **DOPE**. In addition, the imidazolium lipophosphoramidate, (**2**) when combined with co-lipid **3a**, **3d**, **1** or **DOPE,** also produces efficient formulations for mRNA delivery. Regarding the membrane fusion activity in acidic medium of the respective liposomes, it appears that the level of mRNA translation is not always linked only to an endosome leak occurring after the protonation of these co-lipids. When comparing mRNA transfection vs. pDNA transfection, mRNA transfection is higher, with a lower quantity of cationic liposomes. This is probably explained by the presence of denatured mRNA in LXs when the quantity of cationic liposomes increased, as shown by CD for **2**/**DOPE** LX, in contrast to DNA. This study leads to the conclusion that the mRNA/liposomes ratio is a key parameter that must be considered with care. Indeed, the compaction is needed to protect mRNA in the extracellular medium and to favor cell internalization, but a too strong compaction produces conformation changes of mRNA that negatively affects the translation efficacies.

## Figures and Tables

**Table 1:** Physicochemical characterization of **3b**-based liposomes.

Co-Lipids	Mol. Ratio ^£^	Size (nm)	PDI *	ζ Potential (mV)
**3a**	1/1	235 ± 2	0.3	49.4 ± 0.6
**3c**	1/1	102 ± 1	0.2	56 ± 0.5
**3d**	1/1	143 ± 3	0.4	71 ± 1
**3e**	1/1	85 ± 1	0.1	73 ± 0.1
**4a**	1/1	140 ± 2	0.1	72 ± 1
**4b**	1/1	139 ± 2	0.5	67 ± 0.7
**1**	1/1	165 ± 2	0.4	70 ± 0.8
**DOPE 1/1**	1/1	120 ± 1	0.1	79 ± 0.6
**DOPE 2/1**	2/1	116 ± 2	0.3	84 ± 1.1

^£^ Molecular ratio 3b/co-lipid; * PDI stands for the polydispersity index. Values are means ± SD of three different measurements.

**Table 2:** Physicochemical characterization of **2**-based liposomes.

Co-Lipids	Mol. Ratio ^£^	Size (nm)	PDI *	ζ Potential (mV)
**3a**	1/1	101 ± 1	0.5	76 ± 0.7
**3c**	1/1	106 ± 1	0.5	71 ± 0.5
**3d**	1/1	112 ± 2	0.3	71 ± 1.0
**3e**	1/1	117 ± 2	0.2	71 ± 0.9
**4a**	1/1	113 ± 1	0.2	73 ± 1.2
**4b**	1/1	163 ± 2	0.3	53 ± 1.0
**1**	1/1	96 ± 1	0.3	74 ± 0.5
**DOPE 1/1**	1/1	163 ± 1	0.1	67 ± 0.6
**DOPE 2/1**	2/1	122 ± 2	0.1	81 ± 1.1

^£^ Molecular ratio 2/co-lipid; * PDI stands for the polydispersity index. Values are means ± SD of three different measurements.

**Table 3:** Membrane fusion potential of liposomes (%).

	3b-Based Liposomes		2-Based Liposomes	
Co-Lipids	pH 7.4	pH 5.5	pH 7.4	pH 5.5
**3a**	20 ± 1	79 ± 7	0	0
**3c**	0	41 ± 4	0	4.5 ± 0.5
**3d**	0	2.6	0	16 ± 1
**3e**	31 ± 2	21 ± 2	31 ± 3	10 ± 1
**4a**	0	37 ± 3	0	0
**4b**	8 ± 1	0	0	11 ± 1
**1**	34 ± 3	71 ± 6	21 ± 2	97 ± 9
**DOPE 1/1**	1.2	19 ± 2	12 ± 1	58 ± 6
**DOPE 2/1**	0	58 ± 5	0	99 ± 3

Membrane fusion potential was calculated as percent of lipid inter-mixing extent (% fusion) after 15 min. DOTAP/DOPE (1/1) used as a control had fusion of 0 and ~34% at pH 7.4 and pH 5.5, respectively. Values are means ± SD of three different measurements.

**Table 4:** Cell Viability (%) in the presence of mRNA lipoplexes.

Co-Lipids		3b-Based LXs		2-Based LXs	
	**mRNA/L**	**1/0.5**	**1/1**	**1/0.5**	**1/1**
**3a**		94 ± 7	85 ± 7	94 ± 7	95 ± 7
**3c**		93 ± 7	94 ± 8	85 ± 7	42 ± 3
**3d**		88 ± 7	72 ± 5	73 ± 6	69 ± 5
**3e**		95 ± 8	70 ± 5	83 ± 7	84 ± 7
**4a**		76 ± 6	79 ± 6	85 ± 7	100 ± 8
**4b**		93 ± 7	84 ± 7	73 ± 6	57 ± 4
**1**		83 ± 6	100 ± 8	69 ± 5	30 ± 2
**DOPE1/1**		80 ± 6	87 ± 7	92 ± 7	90 ± 7
**DOPE2/1**		84 ± 7	87 ± 7	97 ± 8	89 ± 7

The viability was measured by MTT assay. Viability (%) is absorbance of LX-treated cells/absorbance of control cells × 100. mRNA/L is the ratio of negatively charged mRNA to cationic liposomes. Values are means ± SD of three different experiments.
